# Synthesis, enzyme inhibitory kinetics mechanism and computational study of *N*-(4-methoxyphenethyl)-*N*-(substituted)-4-methylbenzenesulfonamides as novel therapeutic agents for Alzheimer’s disease

**DOI:** 10.7717/peerj.4962

**Published:** 2018-06-26

**Authors:** Muhammad Athar Abbasi, Mubashir Hassan, Aziz-ur-Rehman, Sabahat Zahra Siddiqui, Syed Adnan Ali Shah, Hussain Raza, Sung Yum Seo

**Affiliations:** 1 College of Natural Science, Department of Biological Sciences, Kongju National University, Gongju, South Korea; 2 Department of Chemistry, Government College University, Lahore, Pakistan; 3 Faculty of Pharmacy, Universiti Teknologi MARA, Selangor Darul Ehsan, Malaysia; 4 Atta-ur-Rahman Institute for Natural Products Discovery (AuRIns), Universiti Teknologi MARA, Selangor Darul Ehsan, Malaysia

**Keywords:** Sulfonamides, Molecular docking, Acetylcholinesterase, Alkyl/aralkyl halides, Spectral analysis

## Abstract

The present study comprises the synthesis of a new series of sulfonamides derived from 4-methoxyphenethylamine (**1**). The synthesis was initiated by the reaction of **1** with 4-methylbenzenesulfonyl chloride (**2**) in aqueous sodium carbonate solution at pH 9 to yield *N*-(4-methoxyphenethyl)-4-methylbenzensulfonamide **(3)**.This parent molecule **3** was subsequently treated with various alkyl/aralkyl halides, (**4a–j)**, using *N,N*-dimethylformamide (DMF) as solvent and LiH as activator to produce a series of new *N*-(4-methoxyphenethyl)-*N*-(substituted)-4-methylbenzenesulfonamides **(5a–j)**. The structural characterization of these derivatives was carried out by spectroscopic techniques like IR, ^1^H-NMR, and ^13^C-NMR. The elemental analysis data was also coherent with spectral data of these molecules. The inhibitory effects on acetylcholinesterase and DPPH were evaluated and it was observed that N-(4-Methoxyphenethyl)-4-methyl-N-(2-propyl)benzensulfonamide **(5c)** showed acetylcholinesterase inhibitory activity 0.075 ± 0.001 (IC_50_ 0.075 ± 0.001 µM) comparable to Neostigmine methylsulfate (IC_50_ 2.038 ± 0.039 µM).The docking studies of synthesized ligands **5a–j** were also carried out against acetylcholinesterase (PDBID 4PQE) to compare the binding affinities with IC_50_ values. The kinetic mechanism analyzed by Lineweaver-Burk plots demonstrated that compound (5c) inhibits the acetylcholinesterase competitively to form an enzyme inhibitor complex. The inhibition constants K*i* calculated from Dixon plots for compound **(5c)** is 2.5 µM. It was also found from kinetic analysis that derivative 5c irreversible enzyme inhibitor complex. It is proposed on the basis of our investigation that title compound **5c** may serve as lead structure for the design of more potent acetylcholinesterase inhibitors.

## Introduction

**Figure 1:**
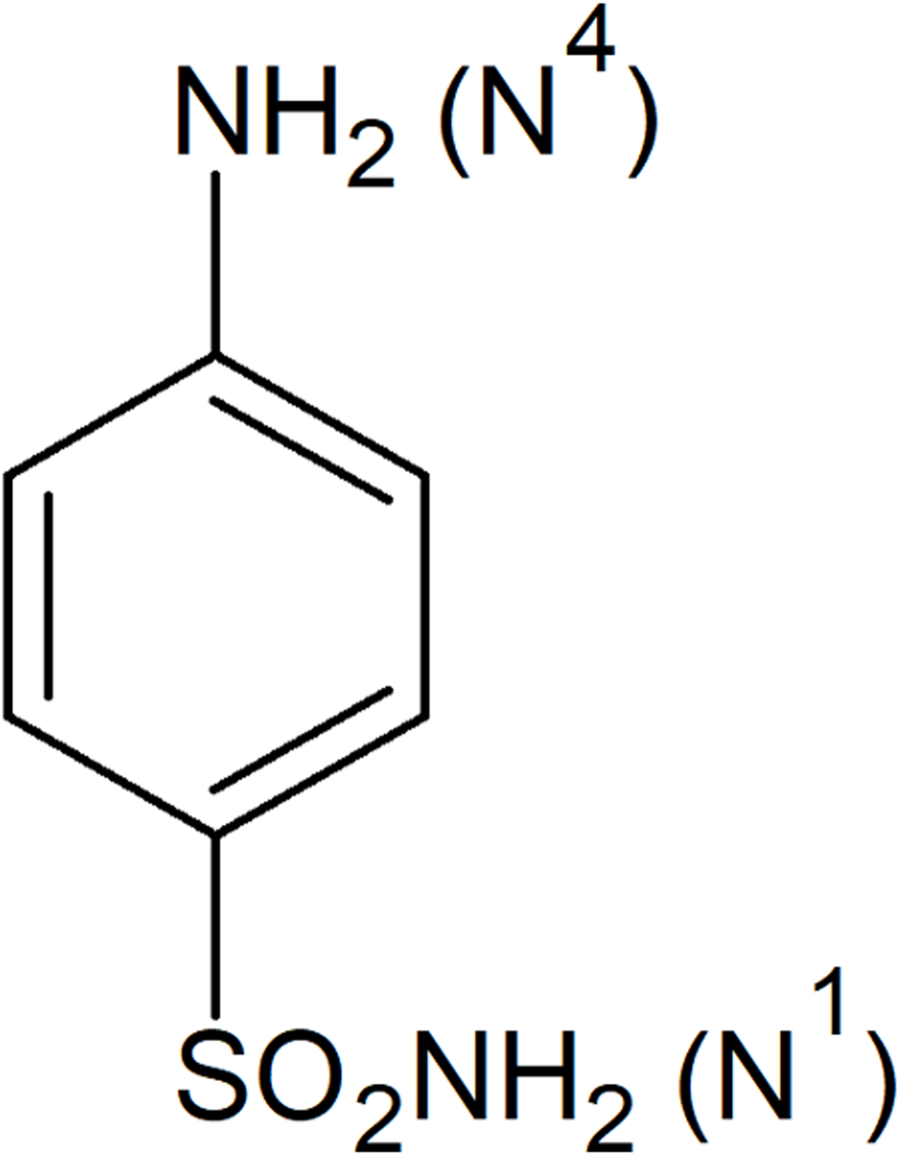
Structure of sulfanilamide.

Sulfonamides are derivatives of sulfonic acids and are the basis of several groups of drugs. The original antibacterial sulfonamides (sulfa drugs) are synthetic. A general method for the synthesis of sulfonamides involves the coupling of sulfonyl chloride with primary or secondary amine or a substituted amine. A sulfonyl group plays a very important role as a key constituent of number of biologically active molecules (Kataoka et al., 1998). Sulfonamides occupy a unique position in the drug industry and exhibit a wide spectrum of biological activities (Shaabani, Soleimani & Rezayan, 2007; Hanson et al., 1990). It has been reported that the antibacterial activity of Prontosil drug was an attribute of the presence of sulfanilamide component (Fouts, Kamm & Brodie, 1957; Neu & Gootz, 1996; Van Meter & Hubert, 2016). The basic structure of sulfanilamide is given in Fig. 1.

The nitrogen of amino group at *para* position is designated as N^4^ while nitrogen of −SO_2_NH_2_ is designated as N^1^. Systemic sulfa drugs are evolved by substitution at N^1^ position whereas gut active sulfa drugs are produced by substituting N^4^ position. Research data showed that by substitution at N^1^ and N^4^ positions about 5,000 compounds have been synthesized which depicts the significance of these positions in designing of novel compounds (Henry, 1943). Several drugs containing sulfonamide functionality are in clinical uses which include antibacterial and antifungal drugs (Zani et al., 2009), anti-inflammatory agents (Dauban & Dodd, 2000), antimigraine agents (Humphrey et al., 1988), anticonvulsant agents (Maryanoff, Nortey & Gardocki, 1987) carbonic anhydrase inhibitors (Supurna et al., 2001; Scozzafava et al., 2000; Maren, 1976), hypoglycemic, protease inhibitors (Roush et al., 1998) and agents acting against diabetic mellitus (Weyer & Hitzel, 1988). They are also found to have extensive applications in cancer chemotherapy (Yoshino et al., 1992).

Acetylcholinesterase (AChE, or acetylhydrolase) is a primary cholinesterase in the body which catalyzes the breakdown of acetylcholine and some other choline esters functioning as neurotransmitters. Acetylcholinesterase belongs to carboxylesterase family of enzymes and its activity serves to terminate synaptic transmission. Cholinestrases are potential target for the symptomatic treatment of Alzheimer’s disease and related dementias (Cygler et al., 1993; Tougu, 2001). Therefore, it is important to search new cholinesterase inhibitors as possible drug candidates (Colovic et al., 2013; Grieg et al., 2014). In our previous attempts, we have reported some sulfonamides as acetylcholinesterase inhibitors and these molecules were having either no substituent or an ethoxy along with halogen substituents in the starting amine (Abbasi et al., 2014a; Abbasi et al., 2014b). In the present investigation, we synthesized a new series of sulfonamides starting from an amine (4-methoxyphenethy amine) bearing an electron donating methoxy group at 4-position in its structure. These new analogues were evaluated for their acetylcholinesterase inhibitory potential and their kinetic study and molecular docking was also performed to establish the binding of these molecules within the active region of target protein.

## Experimental Data

### General

All the chemicals, along with analytical grade solvents, were purchased from Sigma-Aldrich (Darmstadt, Germany), Alfa Aesar (Tewksbury, MA, USA) or Merck (Kenilworth, NJ, USA) through local suppliers. Pre-coated silica gel Al-plates were used for TLC with ethyl acetate and *n*-hexane as mobile phase. Spots were detected by UV_254_.

Gallonkamp apparatus was used to detect melting points in capillary tubes. IR spectra (*ν*, cm^−1^) were recorded by KBr pellet method in the Jasco-320-A spectrometer. ^1^H-NMR spectra (*δ*, ppm) were recorded at 600 MHz (^13^C-NMR spectra, at 150 MHz) in DMSO-d_6_ using the Bruker Advance III 600 Ascend spectrometer using BBO probe. The coupling constant (*J*) is given in Hz and chemical shift (*δ*) in ppm. The abbreviations used in interpretation of ^1^H NMR spectra are as follows: s, singlet; d, doublet; dd, doublet of doublets; t, triplet; br.t, broad triplet; q, quartet; quint, quintet; sex, sextet; sep, septet; m, multiplet.

### Procedure for the preparation of *N*-(4-methoxyphenethyl)- 4-methylbenzensulfonamide (3)

In 250mL round bottom flask, 4-methoxyphenethylamine (2 ml; 0.002 mol; **1**) was added in 40 mL of distilled water at room temperature and solution was stirred for 30 min. Ten percent Aqueous Na_2_CO_3_ solution was added in the reaction mixture to maintain pH up to 8–9. When the mixture was stirred for half an hour, 4-methylbenzenesulfonyl chloride (2.58 g; 0.002 mol; **2**) was added in the reaction mixture gradually. The mixture was stirred again for 2–3 h, and was monitored by TLC until completion in *n*-hexane: ethyl acetate (80:20%; R_f_: 0.7). After completion of reaction, concentrated HCl was added drop wise till pH 5 to obtain the precipitates which was filtered, precipitates were washed with distilled water thoroughly to remove any impurities, and dried to yield the parent molecule, *N*-(4-methoxyphenethyl)-4-methylbenzenesulfonamide (**3**), as off-white powder in 91% yield.

### General Procedure for Synthesis of *N*-(4-methoxyphenethyl)-*N*- (substituted)-4-methylbenzenesulfonamides (5a–j)

*N*-(4-Methoxyphenethyl)-4-methylbenzensulfonamide (0.2 g; 0.065 mmol; **3**) dissolved in 5 mL *N,N*-dimethyl formamide (DMF) was taken in 50 mL round bottom flask. Catalytic amount of lithium hydride (0.065 g; 0.01625 mmol) as an activator was added in the reaction mixture and was stirred for 30 min at room temperature. Then, different alkyl/aralkyl halides (0.065 mmol; **4a–j**) were added into the reaction mixture and was stirred again for 4–5 h. The reaction was monitored by TLC until completion in *n*-Hexane: ethyl acetate (80:20%; Rf: 0.65). When the reaction was completed, iced distilled water was poured into the reaction mixture and was shaken thoroughly till precipitates were formed. Precipitates obtained were filtered, washed and air-dried to get the respective pure products (**5a–j**).

### *N*-Ethyl-*N*-(4-methoxyphenethyl)-4-methylbenzensulfonamide (5a)

Light yellow gel; yield: 72%; Mol. Formula: C_18_H_23_NO_3_S; Mol. Mass: 333 g/mol: iR *υ*: 3,398 (Secondary amide N-H stretching), 3,045 (C-H str. of aromatic ring), 2,856 (C-H str. of aliphatic), 1,536 (C=C aromatic str.), 1,248 (C-O-C stretching of aromatic ether), 1,036 (C-N). ^1^H-NMR (600 MHz, DMSO-*d*_6_): *δ* 7.67 (d, *J* = 8.2 Hz, 2H, H-2′ & H-6′), 7.39 (d, *J* = 7.9 Hz, 2H, H-3′ & H-5′), 7.12 (d, *J* = 8.6 Hz, 2H, H-2 & H-6), 6.85 (d, *J* = 8.6 Hz, 2H, H-3 & H-5), 3.72 (s, 3H, CH_3_-9), 3.24 (br. t, *J* = 7.7 Hz, 2H, CH_2_-8), 3.16 (q, *J* = 7.1 Hz, 2H, CH_2_-1″), 2.71 (br. t, *J* = 7.6 Hz, 2H, CH_2_-7), 2.38 (s, 3H, CH_3_-7′), 1.00 (t, *J* = 7.1 Hz, 3H, CH_3_-2″). ^13^C-NMR (DMSO-*d*_6_, 150 MHz): *δ* 158.32 (C-4), 143.38 (C-4′), 137.15 (C-1′), 130.86 (C-1), 130.22 (C-2 & C-6), 130.12 (C-3′ & C-5′), 127.24 (C-2′ & C-6′), 114.28 (C-3 & C-5), 55.44 (C-9), 49.32 (C-8), 42.96 (C-1″), 34.46 (C-7), 21.37 (C-7′), 14.28 (C-2″). Anal. Calc. for C_18_H_23_NO_3_S (333.15): Calculated: C, 64.84; H, 6.95; N, 4.20. Found: C, 64.79; H, 6.87; N, 4.12.

### *N*-(4-Methoxyphenethyl)-4-methyl-*N*-(1-propyl)benzensulfonamide (5b)

Off-white solid; yield: 75%; m.p.: 79 °C, Mol. Formula: C_19_H_25_NO_3_S; Mol. Mass: 347 g/mol: IR *υ*: 3,399 (secondary amide N-H stretching), 3,085 (C-H str. of aromatic ring), 2,889 (C-H str. of aliphatic), 1,553 (C=C aromatic str.), 1,254 (C-O-C stretching of aromatic ether), 1,050 (C-N). ^1^H-NMR (600 MHz, DMSO-*d*_6_): *δ* 7.68 (d, *J* = 7.9 Hz, 2H, H-2′ & H-6′), 7.40 (d, *J* = 7.9 Hz, 2H, H-3′ & H-5′), 7.11 (d, *J* = 8.3 Hz, 2H, H-2 & H-6), 6.84 (d, *J* = 8.3 Hz, 2H, H-3 & H-5), 3.72 (s, 3H, CH_3_-9), 3.21 (t, *J* = 7.7 Hz, 2H, CH_2_-8), 3.04 (t, *J* = 7.4 Hz, 2H, CH_2_-1″), 2.69 (t, *J* = 7.7 Hz, 2H, CH_2_-7), 2.39 (s, 3H, CH_3_-7′), 1.45 (sex., *J* = 7.3 Hz, 2H, CH_2_-2″), 0.80 (t, *J* = 7.3 Hz, 3H, CH_3_-3″). ^13^C-NMR (DMSO-*d*_6_, 150 MHz): *δ* 158.32 (C-4), 143.40 (C-4′), 137.06 (C-1′), 130.85 (C-1), 130.24 (C-2 & C-6), 130.09 (C-3′ & C-5′), 127.28 (C-2′ & C-6′), 114.31 (C-3 & C-5), 55.48 (C-9), 50.13 (C-1″), 50.02 (C-8), 34.47 (C-7), 21.89 (C-2″), 21.40 (C-7′), 11.45 (C-3″). Anal. Calc. for C_19_H_25_NO_3_S (347.16): Calculated: C, 65.68; H, 7.25; N, 4.03. Found: C, 65.53; H, 7.19; N, 3.95.

### *N*-(4-Methoxyphenethyl)-4-methyl-*N*-(2-propyl)benzensulfonamide (5c)

White solid; yield: 81%; m.p.: 67 °C , Mol. Formula: C_19_H_25_NO_3_S; Mol. Mass: 347 g/mol: IR *υ*: 3,408 (secondary amide N-H stretching), 3,087 (C-H str. of aromatic ring), 2,894 (C-H str. of aliphatic), 1,579 (C=C aromatic str.), 1,258 (C-O-C stretching of aromatic ether), 1,059 (C-N). ^1^H-NMR (600 MHz, DMSO-*d*_6_): *δ* 7.72 (d, *J* = 8.0 Hz, 2H, H-2′ & H-6′), 7.40 (d, *J* = 8.0 Hz, 2H, H-3′ & H-5′), 7.16 (d, *J* = 8.3 Hz, 2H, H-2 & H-6), 6.87 (d, *J* = 8.3 Hz, 2H, H-3 & H-5), 3.96 (sep., *J* = 6.6 Hz, 1H, H-2″), 3.73 (s, 3H, CH_3_-9), 3.18 (br. t, *J* = 7.7 Hz, 2H, CH_2_-8), 2.82 (br. t, *J* = 7.7 Hz, 2H, CH_2_-7), 2.38 (s, 3H, CH_3_-7′), 0.93 (d, *J* = 6.7 Hz, 6H, CH_3_-1″ & CH_3_-3″). ^13^C-NMR (DMSO-*d*_6_, 150 MHz): *δ* 158.34 (C-4), 143.33 (C-4′), 138.20 (C-1′), 131.23 (C-1), 130.23 (C-2 & C-6), 130.20 (C-3′ & C-5′), 127.22 (C-2′ & C-6′), 114.31 (C-3 & C-5), 55.48 (C-9), 49.62 (C-8), 46.34 (C-2″), 37.58 (C-7), 21.39 (C-7′), 20.94 (C-1″ & C-3″). Anal. Calc. for C_19_H_25_NO_3_S (347.16): Calculated: C, 65.68; H, 7.25; N, 4.03. Found: C, 65.56; H, 7.15; N, 3.90.

### *N*-(1-Butyl)-*N*-(4-methoxyphenethyl)-4-methylbenzensulfonamide (5d)

Dull yellow gel; yield: 69%; Mol. Formula: C_20_H_27_NO_3_S; Mol. Mass: 361g/mol: IR *υ*: 3,414 (secondary amide N-H stretching), 3,088 (C-H str. of aromatic ring), 2,898 (C-H str. of aliphatic), 1,584 (C=C aromatic str.), 1,260 (C-O-C stretching of aromatic ether), 1,064 (C-N). ^1^H-NMR (600 MHz, DMSO-*d*_6_): *δ* 7.67 (d, *J* = 8.2 Hz, 2H, H-2′ & H-6′), 7.40 (d, *J* = 8.0 Hz, 2H, H-3′ & H-5′), 7.11 (d, *J* = 8.6 Hz, 2H, H-2 & H-6), 6.84 (d, *J* = 8.6 Hz, 2H, H-3 & H-5), 3.72 (s, 3H, CH_3_-9), 3.22 (t, *J* = 7.7 Hz, 2H, CH_2_-8), 3.06 (t, *J* = 7.5 Hz, 2H, CH_2_-1″), 2.68 (t, *J* = 7.7 Hz, 2H, CH_2_-7), 2.38 (s, 3H, CH_3_-7′), 1.41 (quint., *J* = 7.3 Hz, 2H, CH_2_-2″), 1.23 (sex., *J* = 7.5 Hz, 2H, CH_2_-3″), 0.84 (t, *J* = 7.4 Hz, 3H, CH_3_-4″). ^13^C-NMR (DMSO-*d*_6_, 150 MHz): *δ* 158.33 (C-4), 143.40 (C-4′), 137.01 (C-1′), 130.23 (C-2 & C-6), 130.10 (C-3′ & C-5′), 130.08 (C-1), 127.29 (C-2′ & C-6′), 114.31 (C-3 & C-5), 55.47 (C-9), 50.02 (C-8), 48.18 (C-1″), 34.48 (C-7), 30.66 (C-2″), 21.39 (C-7′), 19.74 (C-3″), 13.97 (C-4″). Anal. Calc. for C_20_H_27_NO_3_S (361.17): Calculated: C, 66.45; H, 7.53; N, 3.87. Found: C, 66.39; H, 7.47; N, 3.75.

### *N*-(4-Methoxyphenethyl)-4-methyl-*N*-(1-pentyl)benzensulfonamide (5e)

Off-white gel; yield: 81%; Mol. Formula: C_21_H_29_NO_3_S; Mol. Mass: 375 g/mol: IR *υ*: 3,417 (secondary amide N-H stretching), 3,090 (C-H str. of aromatic ring), 2,901 (C-H str. of aliphatic), 1,586 (C=C aromatic str.), 1,262 (C-O-C stretching of aromatic ether), 1,067 (C-N). ^1^H-NMR (600 MHz, DMSO-*d*_6_): *δ* 7.67 (d, *J* = 8.2 Hz, 2H, H-2′ & H-6′), 7.39 (d, *J* = 8.0 Hz, 2H, H-3′ & H-5′), 7.10 (d, *J* = 8.6 Hz, 2H, H-2 & H-6), 6.84 (d, *J* = 8.6 Hz, 2H, H-3 & H-5), 3.72 (s, 3H, CH_3_-9), 3.22 (t, *J* = 7.7 Hz, 2H, CH_2_-8), 3.05 (t, *J* = 7.5 Hz, 2H, CH_2_-1″), 2.68 (t, *J* = 7.6 Hz, 2H, CH_2_-7), 2.38 (s, 3H, CH_3_-7′), 1.41 (quint., *J =* 7 .4 Hz, 2H, CH_2_-3″), 1.22 (sex., *J* = 7.2 Hz, 2H, CH_2_-4″), 1.17 (quint., *J* = 7.1 Hz, 2H, CH_2_-2″), 0.81 (t, *J* = 7.3 Hz, 3H, CH_3_-5″). ^13^C-NMR (DMSO-*d*_6_, 150 MHz): *δ* 158.33 (C-4), 143.39 (C-4′), 137.00 (C-1′), 130.87 (C-1), 130.22 (C-2 & C-6), 130.10 (C-3′ & C-5′), 127.29 (C-2′ & C-6′), 114.29 (C-3 & C-5), 55.45 (C-9), 50.03 (C-8), 48.46 (C-1″), 34.51 (C-7), 28.70 (C-3″), 28.18 (C-2″), 22.15 (C-4″), 21.38 (C-7′), 14.24 (C-5″). Anal. Calc. for C_20_H_27_NO_3_S (361.17): Calculated: C, 66.45; H, 7.53; N, 3.87. Found: C, 66.38; H, 7.47; N, 3.74.

### *N*-(1-Heptyl)-*N*-(4-methoxyphenethyl)-4-methylbenzensulfonamide (5f)

Off-white gel; yield: 70%; Mol. Formula: C_23_H_33_NO_3_S; Mol. Mass: 403 g/mol: IR *υ*: 3,419 (secondary amide N-H stretching), 3,093 (C-H str. of aromatic ring), 2,904 (C-H str. of aliphatic), 1,589 (C=C aromatic str.), 1,267 (C-O-C stretching of aromatic ether), 1,069 (C-N). ^1^H-NMR (600 MHz, DMSO-*d*_6_): *δ* 7.66 (d, *J* = 9.9 Hz, 2H, H-2′ & H-6′), 7.38 (d, *J* = 9.7 Hz, 2H, H-3′ & H-5′), 7.10 (d, *J* = 10.3 Hz, 2H, H-2 & H-6), 6.83 (d, *J* = 10.3 Hz, 2H, H-3 & H-5), 3.71 (s, 3H, CH_3_-9), 3.22 (t, *J* = 9.0 Hz, 2H, CH_2_-8), 3.05 (t, *J* = 8.7 Hz, 2H, CH_2_-1″), 2.68 (t, *J* = 9.12 Hz, 2H, CH_2_-7), 2.37 (s, 3H, CH_3_-7′), 1.38 (quint., *J* = 8.1 Hz, 2H, CH_2_-2″), 1.25-1.15 (m, 8H, CH_2_-3″ to CH_2_-6″), 0.84 (t, *J* = 8.46 Hz, 3H, CH_3_-7″). ^13^C-NMR (DMSO-*d*_6_, 150 MHz): *δ* 158.34 (C-4), 143.34 (C-4′), 137.07 (C-1′), 130.86 (C-1), 130.17 (C-2 & C-6), 130.07 (C-3′ & C-5′), 127.27 (C-2′ & C-6′), 114.27 (C-3 & C-5), 55.41 (C-9), 49.99 (C-8), 48.47 (C-1″), 34.54 (C-7), 31.62 (C-5″), 28.72 (C-4″), 28.48 (C-2″), 26.47 (C-3″), 22.45 (C-6″), 21.35 (C-7′), 14.31 (C-7″). Anal. Calc. for C_23_H_33_NO_3_S (403.22): Calculated: C, 68.45; H, 8.24; N, 3.47. Found: C, 68.37; H, 8.17; N, 3.41.

### *N*-(4-Methoxyphenethyl)-4-methyl-*N*-(3-phenyl-1-propyl) benzensulfonamide (5g)

Off-white solid; yield: 80%; m.p.: 73 °C , Mol. Formula: C_25_H_29_NO_3_S; Mol. Mass: 423 g/mol; IR *υ*: 3,434 (Secondary amide N-H stretching), 3,094 (C-H str. of aromatic ring), 2,924 (C-H str. of aliphatic), 1,604 (C=C aromatic str.), 1,266 (C-O-C stretching of aromatic ether), 1,104 (C-N). ^1^H-NMR (600 MHz, DMSO-*d*_6_): *δ* 7.64 (d, *J* = 8.2 Hz, 2H, H-2′ & H-6′), 7.39 (d, *J* = 8.0 Hz, 2H, H-3′ & H-5′), 7.28 (br. t, *J* = 7.6 Hz, 2H, H-3″ & H-5″), 7.15 (br. d, *J* = 7.0 Hz, 2H, H-2″ & H-6″), 7.20 (br. t, *J* = 7.4 Hz, H-4″), 7.09 (d, *J* = 8.6 Hz, 2H, H-2 & H-6), 6.83 (d, *J* = 8.6 Hz, 2H, H-3 & H-5), 3.71 (s, 3H, CH_3_-9), 3.24 (br. t, *J* = 7.6 Hz, 2H, CH_2_-8), 3.10 (br. t, *J* = 7.5 Hz, 2H, CH_2_-9″), 2.68 (br. t, *J* = 7.6 Hz, 2H, CH_2_-7), 2.52 (br. t, *J* = 7.6 Hz, 2H, CH_2_-7″), 2.38 (s, 3H, CH_3_-7′), 1.70 (quint., *J* = 7.4 Hz, 2H, CH_2_-8″). ^13^C-NMR (DMSO-*d*_6_, 150 MHz): *δ* 158.32 (C-4), 143.47 (C-4′), 141.76 (C-1″), 136.86 (C-1′), 130.80 (C-1), 130.25 (C-2 & C-6), 130.11 (C-3′ & C-5′), 128.73 (C-3″ & C-5″), 128.66 (C-2″ & C-6″), 127.30 (C-2′ & C-6′), 126.25 (C-4″), 114.29 (C-3& C-5), 55.45 (C-9), 50.04 (C-8), 48.03 (C-9″), 34.37 (C-7), 32.65 (C-7″), 30.17 (C-8″), 21.40 (C-7′). Anal. Calc. for C_25_H_29_NO_3_S (423.19): Calculated: C, 70.89; H, 6.90; N, 3.31. Found: C, 70.84; H, 6.87; N, 3.25.

### *N*-(4-Methoxyphenethyl)-4-methyl-*N*-(2-methylbenzyl) benzensulfonamide (5h)

Dull white gel; yield: 80%; Mol. Formula: C_24_H_27_NO_3_S; Mol. Mass: 409 g/mol; IR *υ*: 3,465 (secondary amide N-H stretching, 3,097 (C-H str. of aromatic ring), 2,945 (C-H str. of aliphatic), 1,649 (C=C aromatic str.), 1,265 (C-O-C stretching of aromatic ether), 1,159 (C-N). ^1^H-NMR (600 MHz, DMSO-*d*_6_): *δ* 7.79 (d, *J* = 8.5 Hz, 2H, H-2′ & H-6′), 7.45 (d, *J* = 8.5 Hz, 2H, H-3′ & H-5′), 7.34 (br. d, *J* = 7.5 Hz, 1H, H-4″), 7.20 (br. d, *J* = 7.5 Hz, 1H, H-5″), 7.10 (d, *J* = 8.5 Hz, 2H, H-2 & H-6), 6.87 (d, *J* = 8.5 Hz, 2H, H-3 & H-5), 6.80 (br. d, *J* = 8.6 Hz, 1H, H-3″), 6.75 (br. d, *J* = 8.6 Hz, H-6″), 4.31 (s, 2H, CH_2_-7″), 3.67 (s, 3H, CH_3_-9), 3.09 (br. t, *J* = 8.1 Hz, 2H, CH_2_-8), 2.81 (br. t, *J* = 8.1 Hz, 2H, CH_2_-7), 2.41 (s, 3H, CH_3_-7′), 2.32 (s, 3H, CH_3_-8″). ^13^C-NMR (DMSO-*d*_6_, 150 MHz): 158.24 (C-4), 145.65 (C-2″), 143.72 (C-4′), 137.33 (C-1′), 136.68 (C-1″), 130.78 (C-1), 130.36 (C-2 & C-6), 130.18 (C-3″), 130.00 (C-3′ & C-5′), 128.27 (C-6″), 127.11 (C-5″), 126.24 (C-4″), 127.53 (C-2′ & C-6′), 114.28 (C-3 & C-5), 55.42 (C-9), 50.96 (C-7″), 50.12 (C-8), 34.28 (C-7), 21.44 (C-7′), 19.13 (C-8″). Anal. Calc. for C_24_H_27_NO_3_S (409.17): Calculated: C, 70.39; H, 6.65; N, 3.42. Found: C, 70.31; H, 6.57; N, 3.37.

### *N*-(4-Methoxyphenethyl)-4-methyl-*N*-(3-methylbenzyl) benzensulfonamide (5i)

White solid; yield: 80%; m.p.: 30 °C , Mol. Formula: C_24_H_27_NO_3_S; Mol. Mass: 409 g/mol; IR *υ*: 3,465 (secondary amide N-H stretching), 3,096 (C-H str. of aromatic ring), 2,946 (C-H str. of aliphatic), 1,653 (C=C aromatic str.), 1,266 (C-O-C stretching of aromatic ether), 1,164 (C-N). ^1^H-NMR (600 MHz, DMSO-*d*_6_): *δ* 7.81 (d, *J* = 9.9 Hz, 2H, H-2′ & H-6′), 7.45 (d, *J* = 9.9 Hz, 2H, H-3′ & H-5′), 7.23 (br. t, *J* = 9.1 Hz, 1H, H-5″), 7.03 (br. s, 1H, H-2″), 7.11-7.08 (m, 4H, H-2, H-6, H-4″ & H-6″), 6.88 (d, *J* = 9.9 Hz, 2H, H-3 & H-5), 4.29 (s, 2H, CH_2_-7″), 3.69 (s, 3H, CH_3_-9), 3.18 (br. t, *J* = 9.3 Hz, 2H, CH_2_-8), 2.47 (br. t, *J* = 9.3 Hz, 2H, CH_2_-7), 2.40 (s, 3H, CH_3_-7′), 2.26 (s, 3H, CH_3_-8″). ^13^C-NMR (DMSO-*d*_6_, 150 MHz): 158.27 (C-4), 143.61 (C-4′), 137.18 (C-1″), 137.13 (C-1′), 136.70 (C-3″), 130.65 (C-1), 130.36 (C-2 & C-6), 130.32 (C-3′ & C-5′), 130.17 (C-2″), 129.26 (C-4″), 128.26 (C-6″), 127.37 (C-2′ & C-6′),126.31 (C-5″), 114.29 (C-3 & C-5), 55.44 (C-9), 50.51 (C-7″), 49.88 (C-8), 33.96 (C-7), 21.42 (C-7′), 21.30 (C-8″). Anal. Calc. for C_24_H_27_NO_3_S (409.17): Calculated: C, 70.39; H, 6.65; N, 3.42. Found: C, 70.30; H, 6.59; N, 3.39.

### *N*-(4-Methoxyphenethyl)-4-methyl-*N*-(4-methylbenzyl) benzensulfonamide (5j)

White solid; yield: 67%; m.p.: 97 °C , Mol. Formula: C_24_H_27_NO_3_S; Mol. Mass: 409 g/mol; IR *υ*: 3,468 (Secondary amide N-H stretching), 3,096 (C-H str. of aromatic ring), 2,946 (C-H str. of aliphatic), 1,654 (C=C aromatic str.), 1,267 (C-O-C stretching of aromatic ether), 1,167 (C-N). ^1^H-NMR (600 MHz, DMSO-*d*_6_): *δ* 7.80 (d, *J* = 8.2 Hz, 2H, H-2′ & H-6′), 7.73 (d, *J* = 8.1 Hz, 2H, H-3″ & H-5″), 7.45 (d, *J* = 8.2 Hz, 2H, H-3′ & H-5′), 7.41 (d, *J* = 8.1 Hz, 2H, H-2″ & H-6″), 7.15 (d, *J* = 8.5 Hz, 2H, H-2 & H-6), 6.88 (d, *J* = 8.5 Hz, 2H, H-3 & H-5), 4.29 (s, 2H, CH_2_-7″), 3.69 (s, 3H, CH_3_-9), 3.16 (br. t, *J* = 8.2 Hz, 2H, CH_2_-8), 2.82 (br. t, *J* = 8.4 Hz, 2H, CH_2_-7), 2.40 (s, 3H, CH_3_-7′), 2.29 (s, 3H, CH_3_-8″). ^13^C-NMR (DMSO-*d*_6_, 150 MHz): 158.28 (C-4), 143.58 (C-4′), 137.23 (C-1′), 137.17 (C-1″), 136.70 (C-4″), 130.61 (C-1), 130.36 (C-2 & C-6), 130.17 (C-3′ & C-5′), 129.46 (C-3″ & C-5″), 128.27 (C-2″ & C-6″), 127.36 (C-2′ & C-6′), 114.29 (C-3 & C-5), 55.43 (C-9), 50.52 (C-7″), 49.8 (C-8), 33.85 (C-7), 21.56 (C-7′), 21.14 (C-8″). Anal. Calc. for C_24_H_27_NO_3_S (409.17): Calculated: C, 70.39; H, 6.65; N, 3.42. Found: C, 70.29; H, 6.51; N, 3.35.

### *In-vitro* methodology

#### Acetylcholinesterase inhibition assay

The inhibitory activities of synthesized compounds were determined spectrophotometrically using acetylthiocholine iodide as substrate by following the method of (Ellman et al., 1961). Briefly, The assay solution consisted of 180 µL of 50 mM Tris-HCl buffer, pH 7.7, containing (0.1 M sodium chloride and 0.02 M magnesium chloride) and 20 µL of enzyme (AChE, EC 3.1.1.7), acetylcholinesterase (from human erythrocytes, purchased from Sigma-Aldrich, Seoul, Korea) solution (50 U per well); increasing concentrations of test compounds (10 µL) were added to the assay solution and pre incubated for 30 min at 4 °C. After that 5,5′ Dithiobis (2 nitrobenzoic acid) (0.3 mM, 20 µL) and acetylthiocholine iodide (1.8 mM, 20 µL) were added to the reaction mixture and incubated at 37 °C for 10 min, followed by the measurement of absorbance at 412 nm. For non-enzymatic reaction, the assays were carried out with a blank containing all components except acetylcholinesterase. The assay measurements were measured at 412 nm using a micro plate reader (OPTI Max Tunable; Molecular Devices, Sunnyvale, CA, USA) having a wave-length range from 340–850 nm; for 96 well plates. The reaction rates were compared and percent inhibition was calculated due to the presence of tested inhibitors. Neostigmine methylsulfate was used as reference inhibitor. Each concentration was analyzed in three independent experiments run in triplicate. The IC_50_ values were calculated by nonlinear regression using GraphPad Prism 5.0.

The % of Inhibition of Acetylcholinesterase was calculated as following:

\begin{eqnarray*}& \mathrm{Inhibition} (\text{%})=[(B-S)/B] \times  100. \end{eqnarray*}

Here, the *B* and *S* are the absorbance’s for the blank and samples.

#### Determination of AChE inhibition kinetics

The kinetic inhibition of 5c (selected ligand based upon most potent IC_50_ value) was analyzed as the same method described in acetylcholinesterase inhibition assay section. The reaction mixture consisted of 180 µL of 50 mM Tris-HCl buffer (pH 7.7); 10 µL of 5c at the different concentrations (0.00, 0.075 and 0.15 µM) and 20 µL enzyme AChE (50 U per well). We added 20 µL of DTNB 0.3 mM and 20 µL of ATCI substrate at the different concentrations (4, 2, 1, 0.5, 0.25 and 0.125 mM) and mixed, pre-incubated time was same as acetylcholinesterase assay. We measured the absorbance at 412 nm up to 5 min. Lineweaver-Burk plot of the inverse of velocities vs. the inverse of substrate concentration was used for assessment of the type of inhibition AChE kinetically. The EI dissociation constant K*i* was calculated from the secondary plot of 1/V vs. inhibitor concentration. The results (change in absorbance per sec) were processed by using SoftMaxPro software.

### Computational methodology

#### Retrieval of protein structure from PDB

The protein structure of human acetylcholinesterase (PDBID: 4PQE) was accessed form Protein Data Bank (PDB) (https://www.rcsb.org/structure/4pqe). UCSF Chimera 1.10.1 tool was employed for energy minimization by using conjugate gradient algorithm and amber force field (Pettersen et al., 2006). Furthermore, VADAR 1.8 online server was used to interpret the protein architecture of helices, beta-sheets, coils and turns (Willard et al., 2003). The Discovery Studio 2.1 Client was used to view 3D structure of target protein and Ramachandran graph generation (Studio Discovery, 2008).

#### Candidate structures designing in ACD/ChemSketch

The synthesized ligands, **5a–j**, were sketched in ACD/ChemSketch and minimized by UCSF Chimera 1.10.1. The basic biochemical properties and Lipinski’s rule of five (RO5) of synthesized compounds, **5a–j**, were predicted and justified, respectively using online computational tools such as Molinspiration (http://www.molinspiration.com/) and Molsoft (http://www.molsoft.com/).

#### Molecular Docking

Docking experiment was performed on all synthesized compounds (**5a–j**) against targeted protein through PyRx docking tool (Dallakyan & Olson, 2015). In docking experiment, the grid box dimension values were adjusted as *X* =  − 25.27, *Y* = 22.43 and *Z* = 0.665, respectively, with by default exhaustiveness = 8 value. All the compounds **5a–j** were docked separately against crystal structure of human acetylcholinesterase and generated docked complexes were evaluated on the basis of lowest binding energy (Kcal/mol) values and hydrogen/hydrophobic interactions pattern using UCSF Chimera 1.10.1 tool. The 2D graphical depiction of all other docked complexes were evaluated by Discovery Studio tool.

## Results and Discussion

### Chemistry

**Figure 2:**
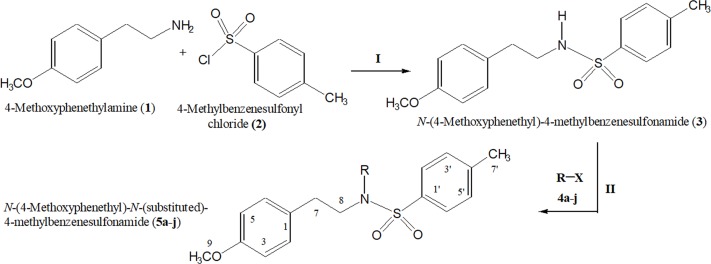
Outline for the synthesis of different *N*-substituted derivatives, **5a–j**, of *N*-(4-methoxyphenethyl)-4-methylbenzensulfonamide (**3**). Reagents & Conditions: (I) Aq. Na_2_CO_3_ soln./pH 9–10/stirring at RT for 2–3 h. (II) DMF/LiH/stirring at RT for 0.5 h for activation/then addition of R-X (**4a–j**) and stirring finally for 4–5 h.

In the presented work, 10 sulfonamide derivatives, (**5a–j**), were synthesized with 4-methoxyphenethylamine (**1**) as starting material according to the outline illustrated in Fig. 2 with various substituents listed in Table 1. The procedures and conditions of the reactions are discussed in the experimental section in multistep reactions. The first step involved the reaction of 4-methoxyphenethylamine (**1**) and 4-methylbenzenesulfonyl chloride (**2**) in aqueous alkaline medium with 4–5 h stirring at room temperature to afford parent molecule; *N*-(4-methoxyphenethyl)-4-methylbenzensulfonamide **(3**), which was isolated by acidification of the reaction mixture to pH 2–3 with concentrated HCl in good yield as off-white powder. In the second step **3**, was subjected to nucleophilic substitution using different alkyl/aralkyl halides (**4a**–**j;** one in each reaction) as electrophiles in polar aprotic solvent, i.e., DMF, using LiH as a base to achieve target *N*-(4-methoxyphenethyl)-4-methyl-*N*-(substituted)benzensulfonamides **(5a–j)**. These synthesized derivatives were subjected to structural analysis using spectroscopic techniques like IR, ^1^H-NMR and ^13^C-NMR, along with elemental analysis. The structural characterization of one of the molecules is discussed hereby in detail for the expediency of the readers. The molecule **5b** was obtained as white solid in 90.3% yield having melting point 99 °C. The molecular formula of this compound was established by counting the number of protons in its ^1^H-NMR spectrum and number of carbon resonances in ^13^C-NMR spectrum. The CHNS analysis data was also in agreement with its molecular formula, C_19_H_25_NO_3_S. Various functionalities in the molecule were affirmed by its IR data. Absorption bands were observed at 3,399 (secondary amide N-H stretching), 3,085 (C-H stretching of aromatic ring), 2,889 (C-H stretching of aliphatic), 1,553 (C=C aromatic stretching), 1,254 (C-O-C stretching of aromatic ether), 1,050 (C-N). In its ^1^H-NMR spectrum (Figs. S1–S3), the presence of 4-methylbenzenesulfonyl moiety was ascertained by an A_2_B_2_ spin system in aromatic region represented by two *ortho*-coupled doublets at *δ* 7.68 for 2H, positioned at H-2′ & H-6′, and *δ* 7.40, 2H, for H-3′ & H-5′, along with a singlet of methyl group in aliphatic region at *δ* 2.39 (s, 3H, CH_3_-7′). Similarly, another *di*-*ortho* coupled pattern was observed for 4-methoxyphenethylamino moiety at *δ* 7.11 (d, *J* = 8.3 Hz, 2H, H-2 & H-6) and *δ* 6.84 (d, *J* = 8.3 Hz, 2H, H-3 & H-5), along with a singlet at *δ* 3.72 for –OCH_3_ group at 9 position. Triplets were observed for two connected methylenes, resonating one at *δ* 3.21 (CH_2_-8) and other at *δ* 2.69 (CH_2_-7). The chemical shifts of these were consistent that former methylene was attached with a nitrogen atom while latter was linked with an aromatic ring. The 1-Propyl group, substituted at nitrogen atom, displayed three signals, a triplet at *δ* 3.04 (CH_2_-1″), a sextet at *δ* 1.45 for CH_2_-2″ and a triplet for terminal methyl group at *δ* 0.80 (CH_3_-3″). The ^13^C-NMR spectrum (Fig. S4) also supported the formation of product as characteristic signals were displayed for aforementioned moieties. 4-Methylbenzenesulfonyl group was embodied by resonances at *δ* 143.40 (C-4′), 137.06 (C-1′), 130.09 (C-3′ & C-5′) and 127.28 (C-2′ & C-6′) along with a signal of *para*-positioned methyl group at *δ* 21.40 (C-7′). Similarly, the signals of 4-methoxyphenethylamino unit were pragmatic at *δ* 158.32 (C-4), 130.85 (C-1), 130.24 (C-2 & C-6), 114.31 (C-3 & C-5), 55.48 (C-9), 50.02 (C-8) and 34.47 (C-7). The 1-Propyl substituent attached to the nitrogen atom in the molecule was corroborated by three peaks at *δ* 50.13 (C-1″), 21.89 (C-2″) and 11.45 (C-3″). So, on the basis of aforementioned cumulative spectral evidences, the structure of **5b** was confirmed and it was named as *N*-(4-methoxyphenethyl)-4-methyl-*N*-(1-propyl)benzensulfonamide. A similar protocol was exercised for the structural characterization of other derivatives.

**Table 1:** Different alkyl/aralkyl substituents (−*R*) in **4a-j** and **5a-j**.

Compound	−*R*	Compound	−*R*
**4a, 5a**	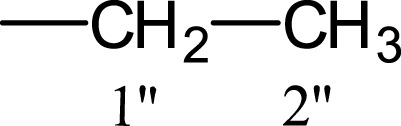	**4f, 5f**	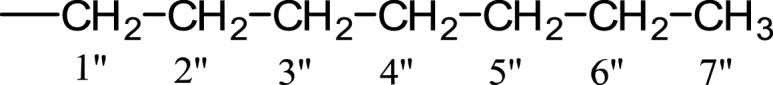
**4b, 5b**	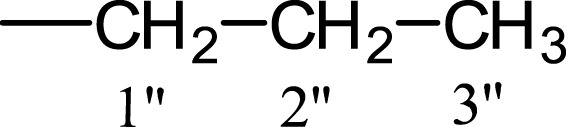	**4g, 5g**	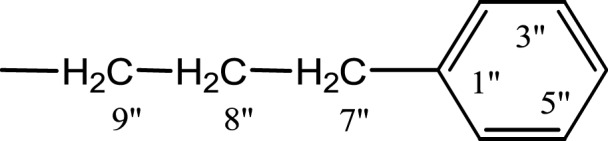
**4c, 5c**	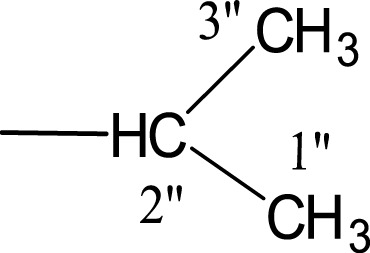	**4h, 5h**	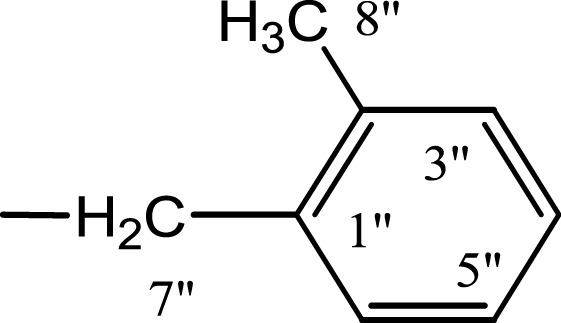
**4d, 5d**	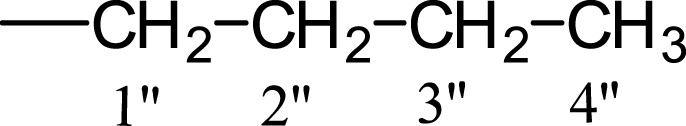	**4i, 5i**	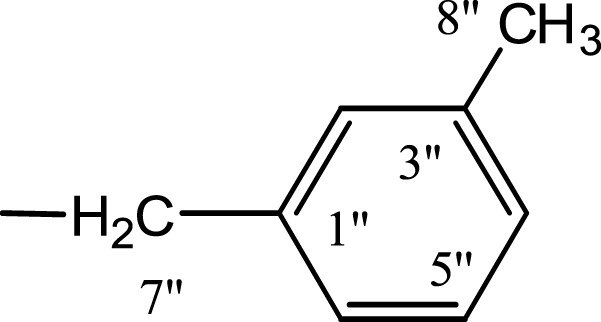
**4e, 5e**	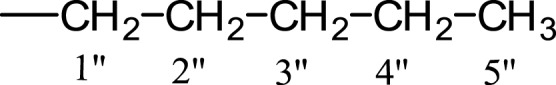	**4j, 5j**	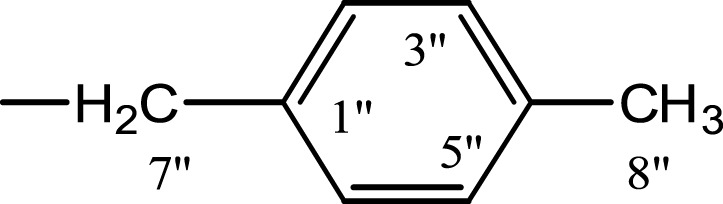

### Acetylcholinesterase inhibition and structure–activity relationship

Various *N*-(4-Methoxyphenethyl)-*N*-(substituted)-4-methylbenzenesulfonamides (**5a–j**) have been designed to evaluate their inhibitory effects on acetylcholinesterase enzyme. Neostigmine methylsulfate, which is a competitive acetylcholinesterase inhibitor, was used as standard for comparison purpose. The targeted sulfonamides as described in the preceding section are of keen interest because most of the molecules exhibited lower IC_50_ values as compared to the standard used. The most active compound was **5c** which exhibited better IC_50_ value of 0.075 ± 0.001 µM, relative to the standard having an IC_50_ value of 2.038 ± 0.039 µM. The inhibitory potential of **5c** might be attributed to the substitution of a branched alkyl (isopropyl) group in this molecule. Similarly, the molecules **5b** and **5d** also exhibited good inhibitory potentials against acetylcholinesterase with IC_50_ values of 0.119 ± 0.701 µM and 0.124 ± 0.021 µM, respectively. The suitable inhibitory potential of **5b** and **5d** might be an outcome of the substitution of 1-propyl and 1-butyl groups, respectively. Therefore, it has been exposed from our bioassay results (Table 2) that the *N*-substitution with a medium sized alkyl group could render better activities in these molecules.

**Table 2:** Acetylcholinesterase (from human erythrocytes) inhibitory activity (5a-5j).

Compound	Acetylcholinesterase IC_50_ ± SEM (µM)
5a	22.549 ± 1.2343
5b	4.1643 ± 0.3179
5c	0.0751 ± 0.0189
5d	0.1195 ± 0.0102
5e	0.3979 ± 0.0123
5f	0.4444 ± 0.0150
5g	0.7980 ± 0.0138
5h	1.0108 ± 0.0053
5i	0.4695 ± 0.0109
5j	1.1077 ± 0.0999
Neostigmine methylsulfate	2.0366 ± 0.0581

**Notes.**

Values are expressed as mean ± SEM.

SEM Standard Error of the Mean

### Kinetics mechanism

**Figure 3:**
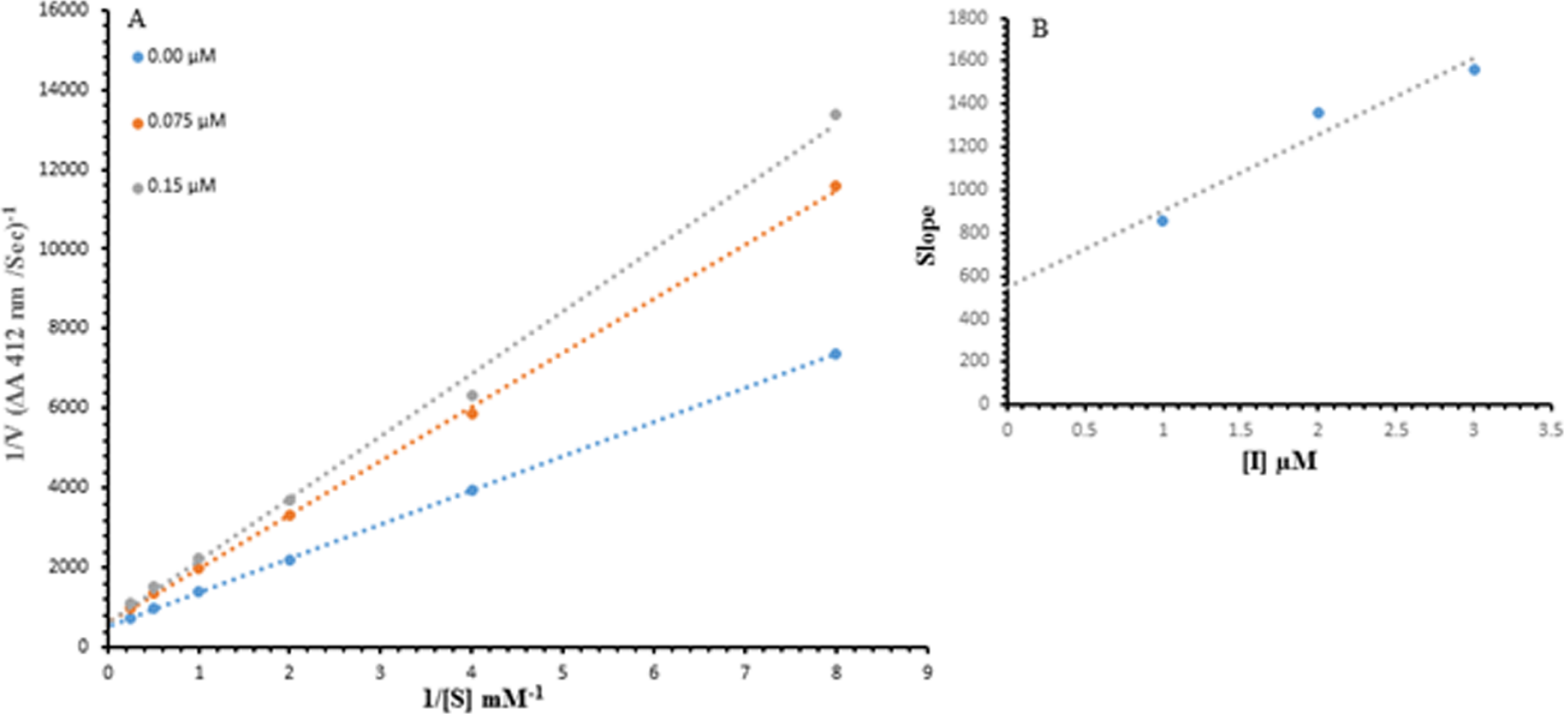
Lineweaver–Burk plots. Lineweaver–Burk plots for inhibition of acetylcholine esterase from human erythrocytes in the presence of Compound 5c (A). Concentrations of 5c were 0.00, 0.075 and 0.15 µM; substrate acetylthiocholine iodide concentrations were 4, 2, 1, 0.5, 0.25, and 0.125 mM. (B) The inset represents the plot of the slope.

To understand the inhibitory mechanism of synthetic compound (**5c**) against acetylcholinesterase was analyzed using kinetic assay. Based upon our IC_50_ results, we select our most potent compound **5c** to determine their inhibition type and inhibition constant. The kinetic results of the enzyme by the Lineweaver-Burk plot of 1/V versus substrate acetylthiocholine iodide 1/[S] in the presence of different inhibitor concentrations gave a series of straight lines, the result of Lineweaver-Burk plot of compound **5c** showed that *V*_max_ remains the same without significantly effecting the slopes. *K*_*m*_ increases with increasing concentration while *V*_max_ remains the same with insignificant difference. This behavior indicates that **5c** compound inhibits the enzyme in competitive manner (Fig. 3A). Second plot (Fig. 3B) of slope against concentration of **5c** showed EI dissociation constant. *K*_*i*_ was calculated from inhibitor concentration of **5c** versus the slope and *K*_*i*_ was found to be 2.5 µM.

### Computational analysis

#### Human acetylcholinesterase structural evaluation

**Figure 4:**
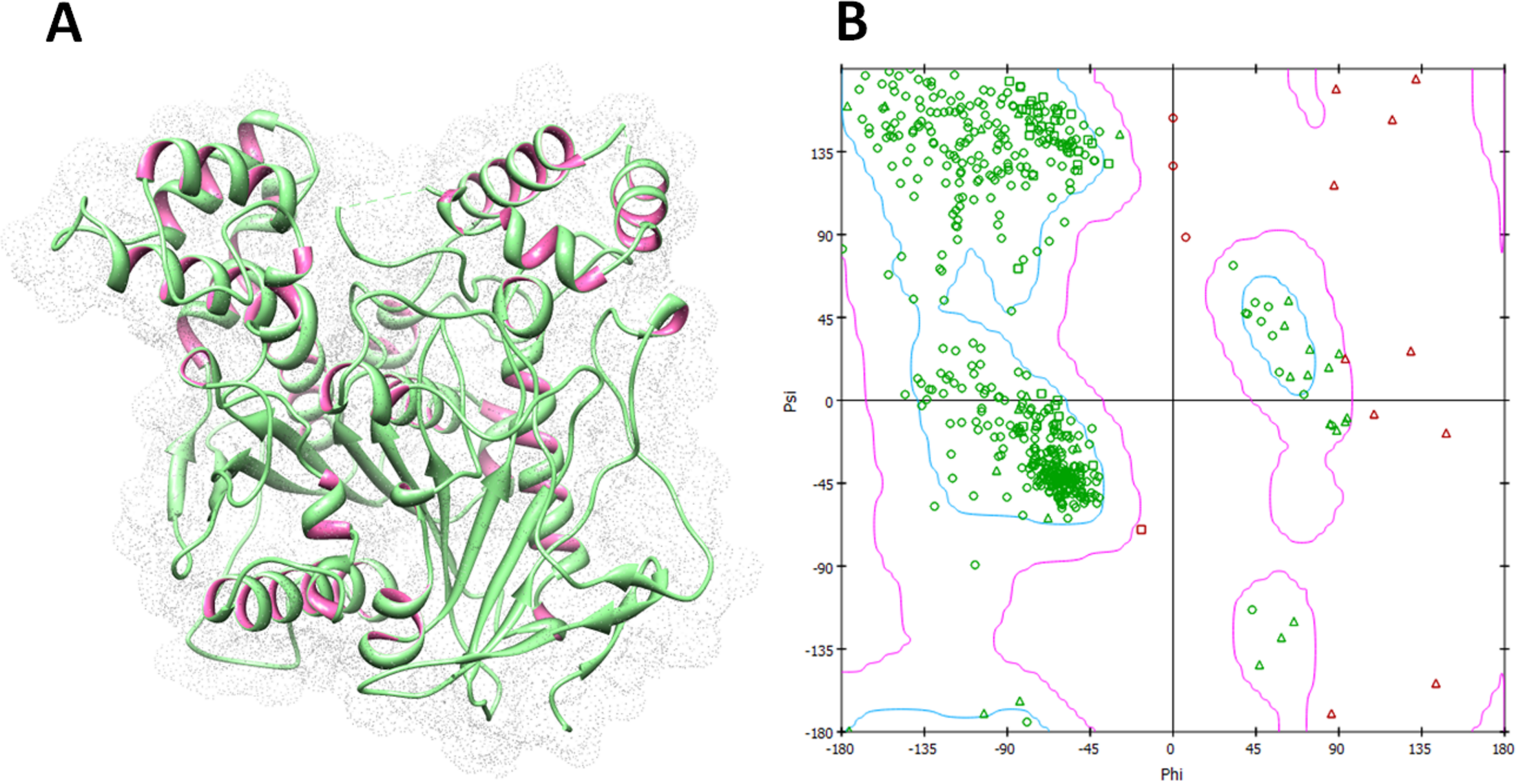
Human acetylcholinesterase and Ramachandran graph. (A) Protein structure of human acetylcholinesterase; (B) Ramachandran graph of target protein.

Human acetylcholinesterase is a class of hydrolase protein having single chain and comprises 543 amino acids. The VADAR 1.8 structure analysis of human acetylcholinesterase depicted that, it consists of 33% *α*-helices, 24% *β*-sheets, 41% coils and 21% turns. The X-ray diffraction study confirmed its resolution 2.9Åand unit cell crystal dimensions. The unit cell coordinates length values were observed for *a* = 125.31, *b* = 125.31 and *c* = 131.40 with angles 90°, 90° and 120° for all *α*, *β* and *γ* dimensions, respectively. The Ramachandran plots and values indicated that 93.50% of protein amino acids were existed in favored region and 99% residues were lie in allowed region (Figs. 4A, 4B). The Ramachandran graph values showed the good accuracy of phi (*φ*) and psi (*ψ*) angles among the coordinates of receptor and most of residues were plunged in acceptable region.

#### Bio-chemical properties and Lipinski’s rule of five (RO5) validation

The biochemical properties of all the synthesized compounds (**5a–j**) were predicted by using computational tools. The synthesized compounds were validated through RO5 analysis. It has been observed that log *P* and molecular mass values should be less than 5 and 500 (g/mol), respectively. Moreover, compounds should possess no greater than 10 HBA and 5 HBD, respectively. Literature data exposed that the exceed values of HBA and HBD results in poor permeation (Kadam & Roy, 2007). The hydrogen-bonding capacity has been considered as significant parameter for drug permeability. Our results justified that the all synthesized compounds possess <10 HBA and <5 HBD values which were comparable with standard values. However, log *P* values of 5d–j are slightly greater than standard value (>5) (Table 3). However, multiple examples are available for RO5 violation amongst the existing drugs (Bakht et al., 2010; Tian et al., 2015).

**Table 3:** Biological properties of synthesized compounds.

Properties	5a	5b	5c	5d	5e	5f	5g	5h	5i	5j
Mol.weight (g/mol)	333.14	347.16	347.16	316.17	375.19	403.22	423.19	409.17	409.17	409.17
No. HBA	4	4	4	4	4	4	4	4	4	4
No. HBD	0	0	0	0	0	0	0	0	0	0
Mol. Log*P*	4.41	4.89	4.75	5.37	5.85	6.82	6.34	5.66	5.78	5.78
SC	0	0	0	0	0	0	0	0	0	0
Mol. Vol (A^3^)	324.38	342.48	340.29	360.38	378.29	414.10	414.42	398.62	399.61	399.53
MolPSA (A^2^)	38.86	39.11	39.92	39.11	39.11	39.11	38.84	38.86	38.86	38.86
Drug likeness Score	−0.24	−0.41	−0.59	−0.41	−0.61	−0.61	−0.38	−0.11	−0.31	−0.46

**Notes.**

HBA Hydrogen Bond Acceptor HBD Hydrogen Bond Donor SC No of stereo centers

**Figure 5:**
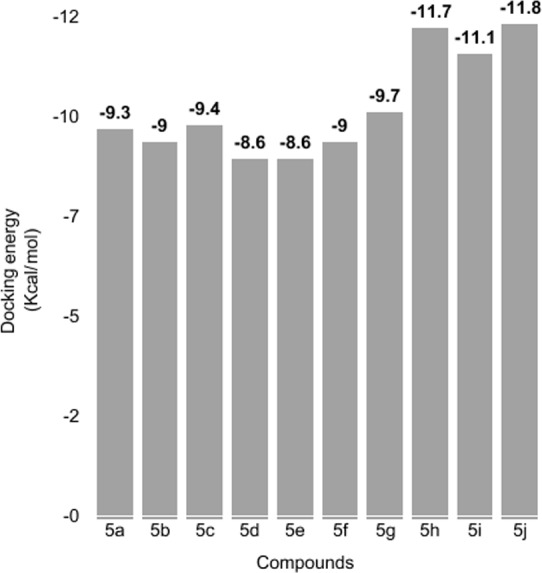
Docking energy. Docking energy values of all synthesized docked complexes.

**Figure 6:**
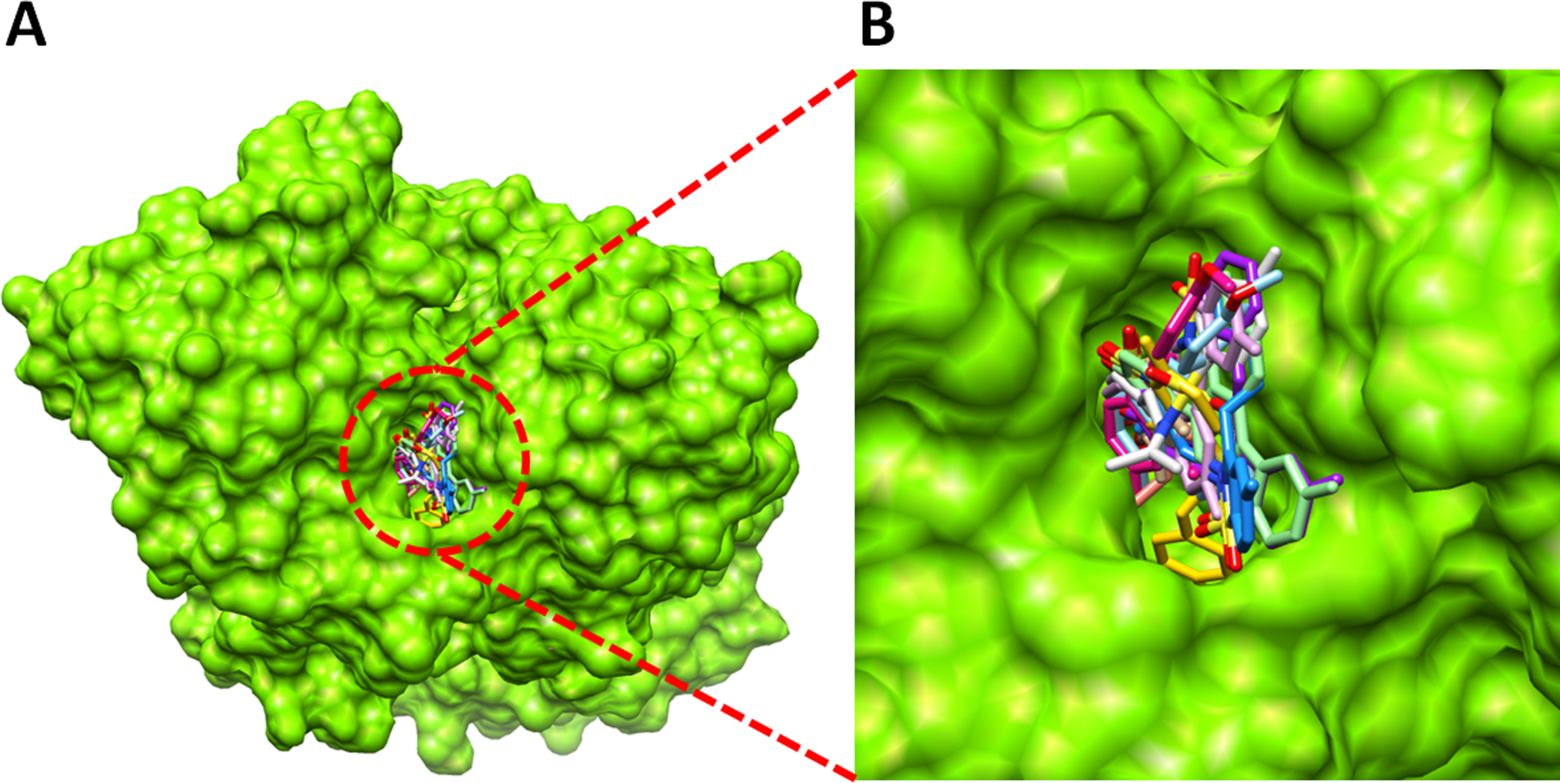
Binding pocket of target protein. (A) Binding pocket of target protein (B) Closer view of ligands structure inside the receptor molecule.

#### Molecular docking analyses

The docked complexes of synthesized compounds (**5a–j**) were analyzed on the basis of lowest binding energy values (Kcal/mol) and hydrogen/hydrophobic bonding analyses. The docking energy values and their binding pattern inside the active region of target protein is mentioned in Figs. 5 and 6 (A, B), respectively. Docking energy results showed that 5h–j were the most active compounds with best binding energy value (−11.70, −11.10 and −11.80 Kcal/mol) compared to others derivatives. Moreover, both 5d-e docked complexes showed lowest energy value (−8.60 Kcal/mol). The compounds 5a-c also showed good energy values. The docking energy values of all the docking complexes was calculated by using Eq. (1).

(1) \begin{eqnarray*}& \Delta \text{Gbinding}=\Delta \text{Ggauss}+\Delta \text{Grepulsion}+\Delta \text{Ghbond}+\Delta \text{Ghydrophobic}+\Delta \text{Gtors}.\end{eqnarray*}

Here, ΔGgauss: attractive term for dispersion of two gaussian functions, ΔGrepulsion: square of the distance if closer than a threshold value, ΔGhbond: ramp function—also used for interactions with metal ions, ΔGhydrophobic: ramp function, ΔGtors: proportional to the number of rotatable bonds. The standard error for Autodock is reported as 2.5 Kcal/mol. However, all the synthesized compounds have no more than standard docking energy value difference.

#### Structure activity relationship (SAR) analyses between 5c and target protein

**Figure 7:**
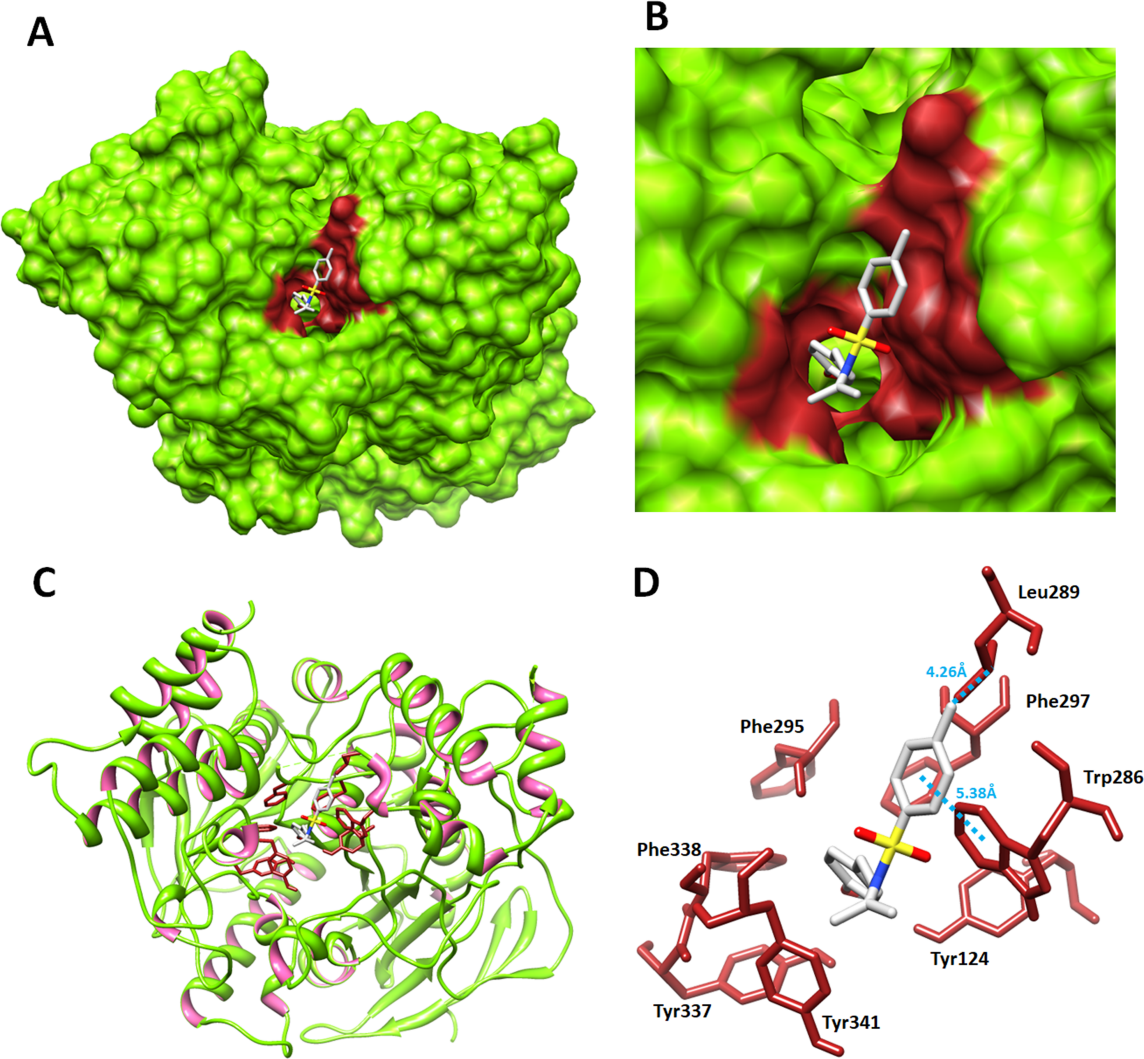
Molecular docking interaction of 5c with acetylcholinesterase. (A) The general overview of docking depiction. The protein structure is represented in green color in surface format while ligand is highlighted in grey color. (B) The closer view of binding pocket interaction with best conformation position of ligand against target protein. The ligand molecule is depicted in grey color while their functional groups such as oxygen, amino and sulfur are showed in red, blue and yellow colors, respectively. (C) The docking complex is represented with ligand conformation. Amino acids are highlighted in dark brown color, while protein structure is represented in green and pink colors, respectively. (D) The closer view of docking complex. The residues involved in the interaction pattern are highlighted in maroon. Light blue dotted lines with distance mentioned in angstrom (Å) are justified for hydrophobic interactions.

All synthesized compounds directly interact within the active region with different conformational positions. Based on *in-vitro* results 5c was most active compounds enzyme inhibition experiment. Therefore, 5c was most active in the *in-vitro* analysis therefore, selected to view conformational pose in the target protein. The SAR analysis shows that 5c interacted with protein residues Leu289 and Trp286 by hydrophobic and *π*–*π* interaction, respectively. The methyl group of benzene ring form hydrophobic interaction against Leu289 having bond length 4.26 Å. Moreover, single *π*–*π* interaction was observed between benzene of 5c and Trp286 residue having distance 5.38 Å. Literature study also justified that these interacted residues are significant in downstream signaling pathways and justify the significance of our docking results (Fang et al., 2014; Simeon et al., 2016). The graphical depiction of 5c docking complex is mentioned in Figs. 7A–7D. However, binding pocket and all the other docked complexes are mentioned in Figs. S5–S13.

## Conclusion

In this research article, a novel series of sulfonamides derived from 4-methoxyphenethylamine were synthesized and the synthesized compounds were characterized through FT-IR, 1H NMR, 13C NMR. All the synthesized compounds showed significant activity against acetylcholinesterase. Kinetic studies were explored to find the binding mode of inhibition, and it was found that compound **5c** inhibits acetylcholinesterase via competitive inhibition mode having a K*i* value 2.5 µM. Molecular docking studies also found in good correlation with the experimental results. Both bioactivity and computational studies results depicted that these newly synthesized molecules can serve a structural template in designing novel drugs against Alzheimer’s disease.

##  Supplemental Information

10.7717/peerj.4962/supp-1 Supplemental Information 1 Supplementary files

10.7717/peerj.4962/supp-2 Data S1 IC 50 data

10.7717/peerj.4962/supp-3 Data S2 Kinetics data

10.7717/peerj.4962/supp-4 Supplemental Information 2 Scheme

10.7717/peerj.4962/supp-5 Data S3 Raw data for Table 2

10.7717/peerj.4962/supp-6 Supplemental Information 3 NMR data

## Additional Information and Declarations

### Data Availability

The following information was supplied regarding data availability: The raw data are provided in the Supplemental Files.
