# Supplementary material for: Synthesis, enzyme inhibitory kinetics mechanism and computational study of N-(4-methoxyphenethyl)-N-(substituted)-4-methylbenzenesulfonamides as novel therapeutic agents for Alzheimer’s disease
Source: PeerJ. 2018 Jun 26;6:e4962. doi: 10.7717/peerj.4962 (PMC6025150; doi:10.7717/peerj.4962)
Supplement: Supplemental Information 1 [file peerj-06-4962-s001.docx]

**Supplementary Data**

**Synthesis, enzyme inhibitory kinetics mechanism and computational study of *N*-(4-methoxyphenethyl)-*N*-(substituted)-4-methylbenzenesulfonamides as novel therapeutic agents for alzheimer’s disease**

Muhammad Athar Abbasi^1,*^, Mubashir Hassan^2^, Aziz-ur-Rehman^1^, Sabahat Zahra Siddiqui^1^, Syed Adnan Ali Shah^3,4^, Hussain Raza^2^ and Sung-Yum Seo^2,*^

*^1^Department of Chemistry, Government College University, Lahore-54000, Pakistan.*

*^2^College of Natural Sciences, Department of Biological Science, Kongju National University, Gongju, 32588, South Korea.*

*^3^Faculty of Pharmacy & ^4^Atta-ur-Rahman Institute for Natural Products Discovery (AuRIns), Level 9, FF3, Universiti Teknologi MARA, Puncak Alam Campus, 42300 Bandar Puncak Alam, Selangor Darul Ehsan, Malaysia.*


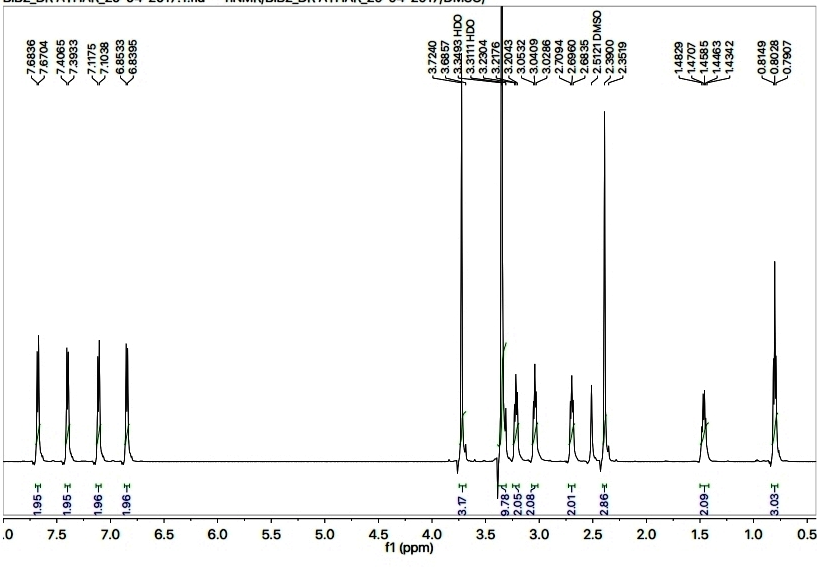


**Figure S1:** ^1^H-NMR spectrum of **5b**


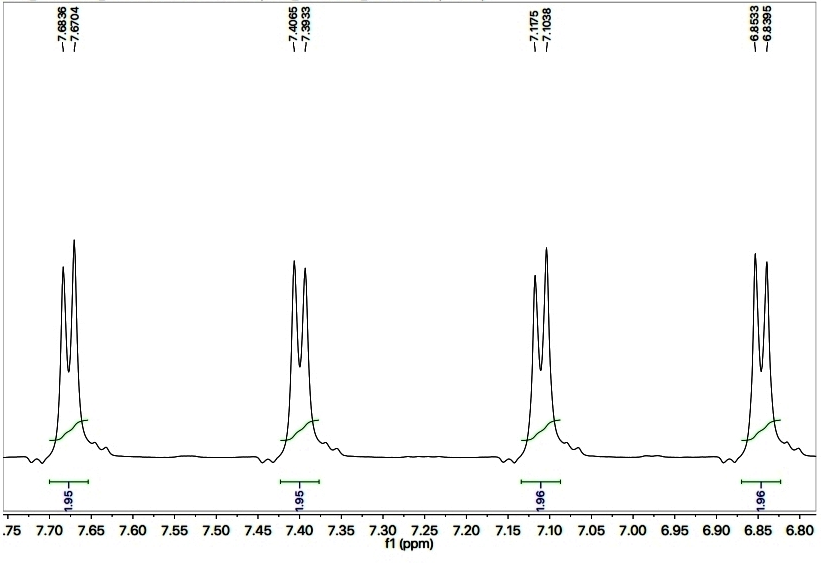


**Figure S2:** Aromatic region of ^1^H-NMR spectrum of **5b**.


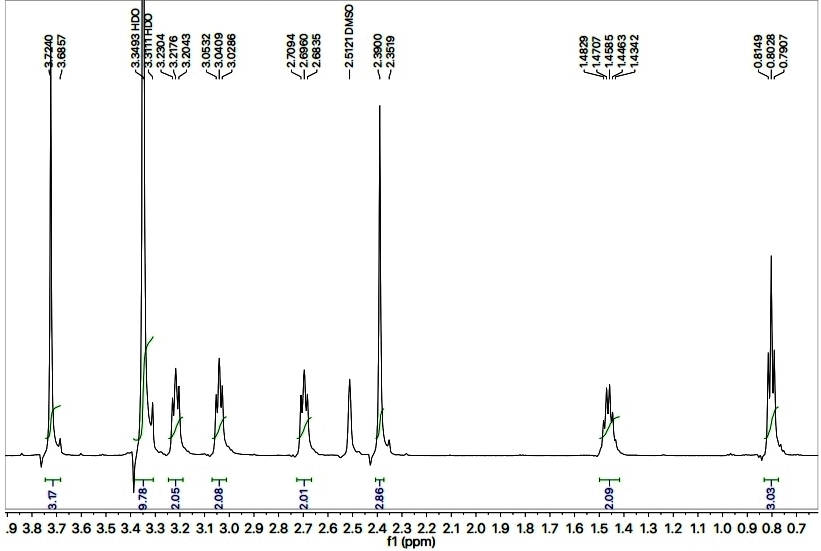


**Figure S3:** Aliphatic region of ^1^H-NMR spectrum of **5b**.


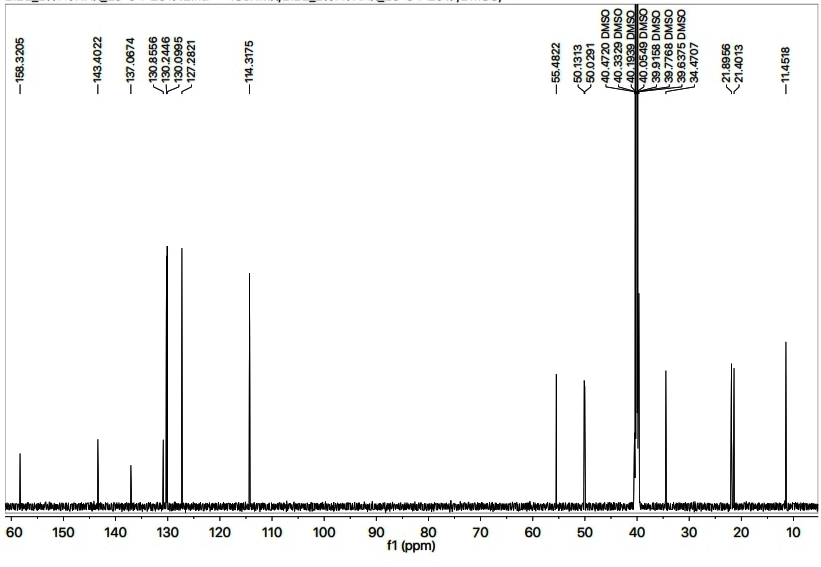


**Figure S4:** ^13^C-NMR spectrum of **5b**.


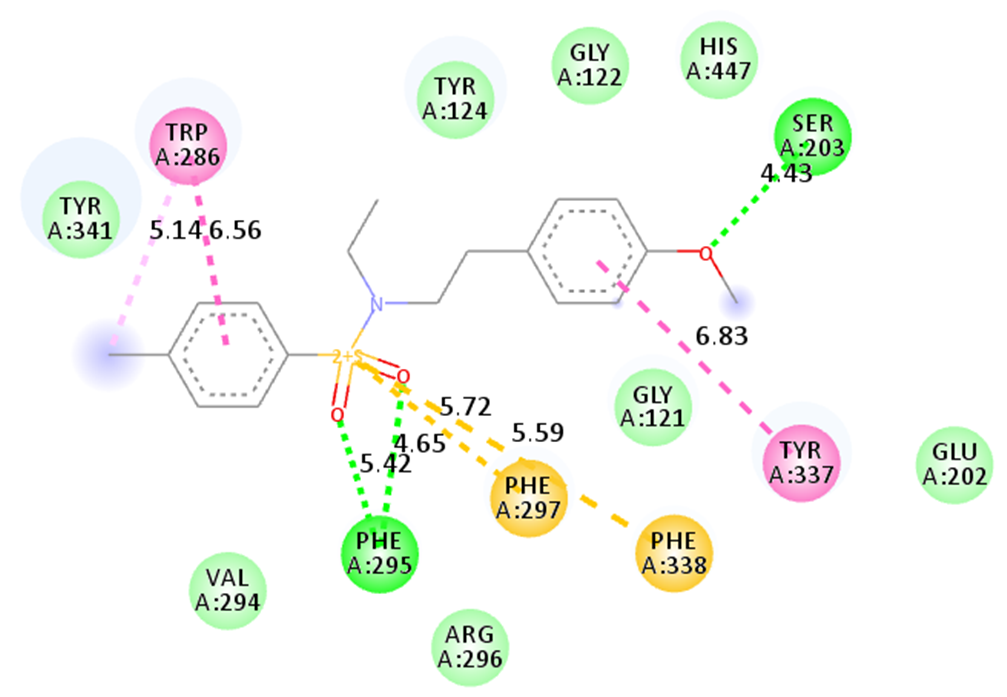


**Figure S5:** Docking complex of 5a


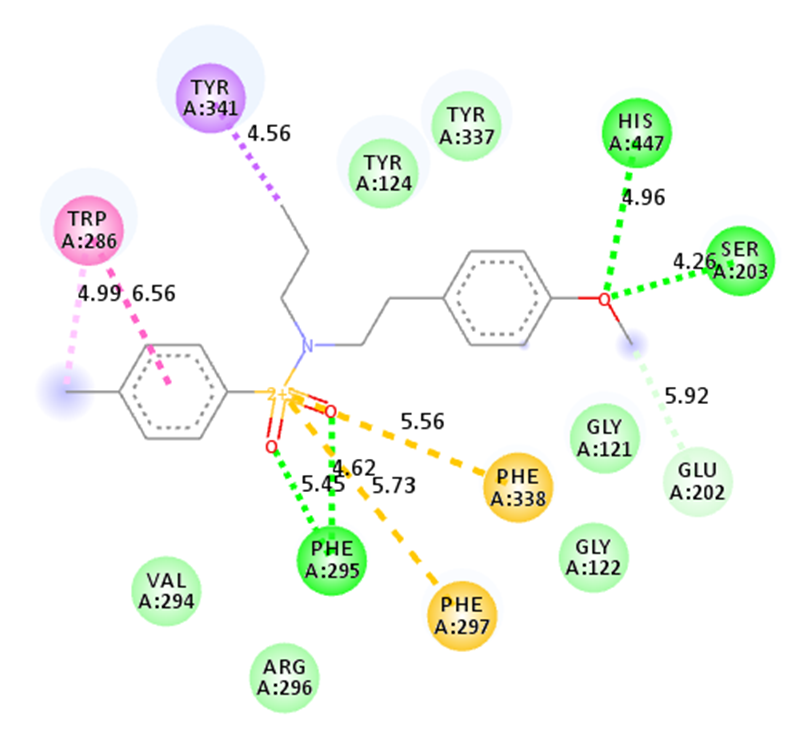


**Figure S6:** Docking complex of 5b


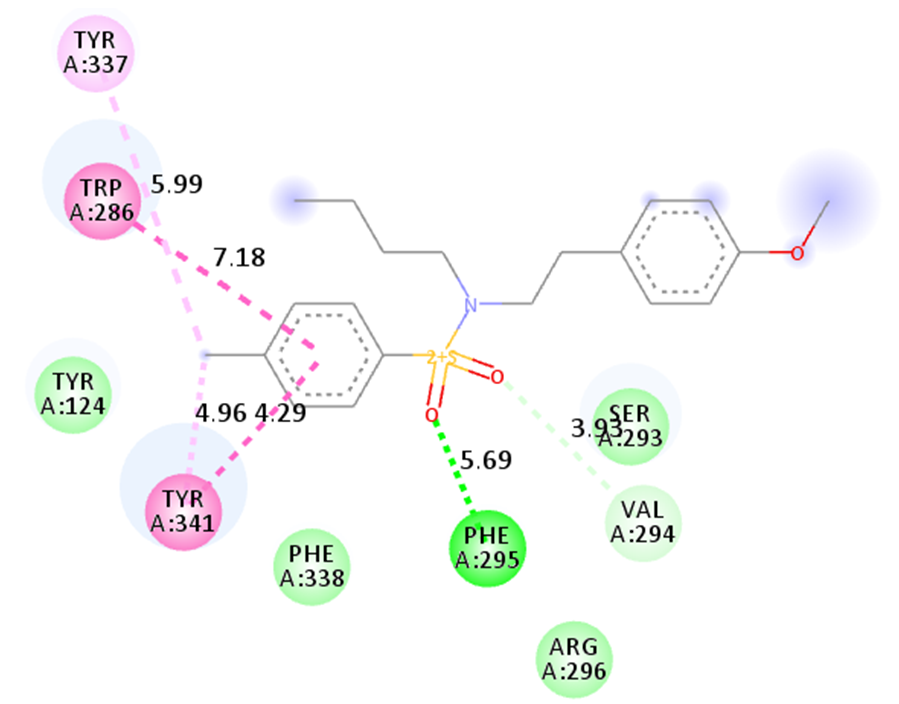


**Figure S7:** Docking complex of 5d


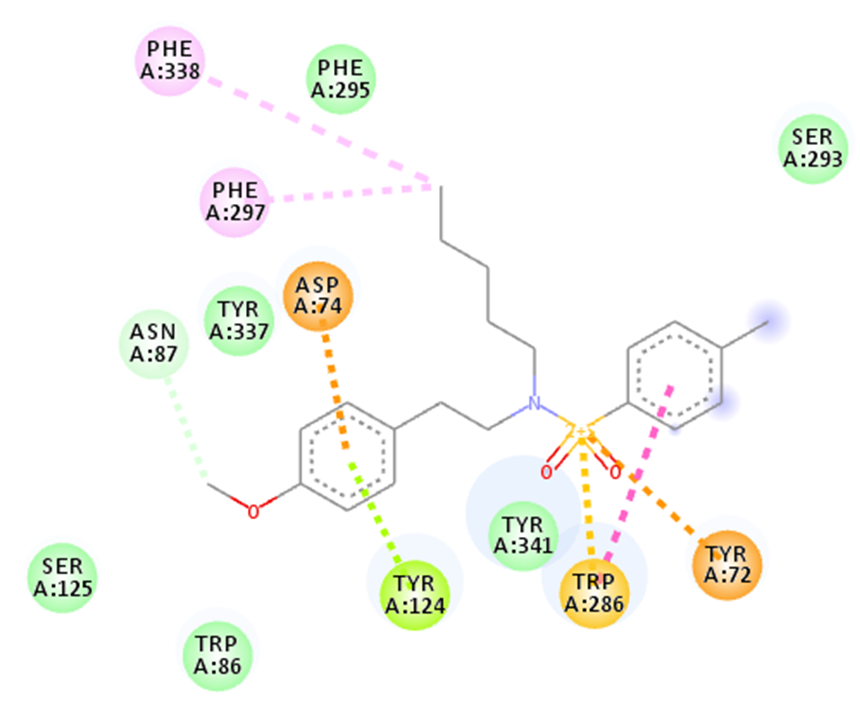


**Figure S8:** Docking complex of 5e


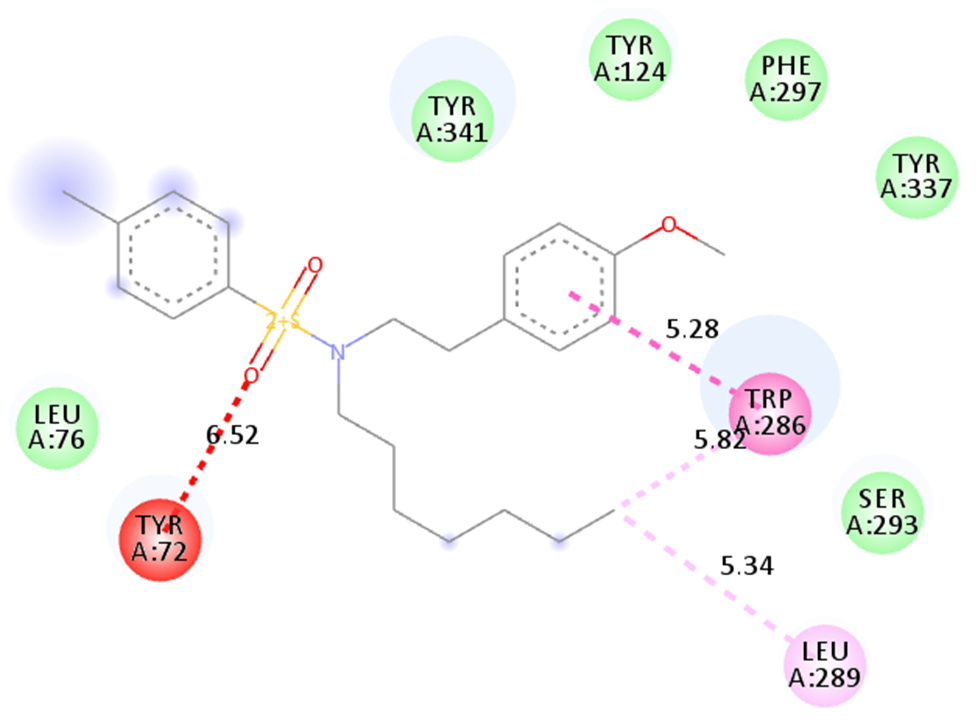


**Figure S9:** Docking complex of 5f


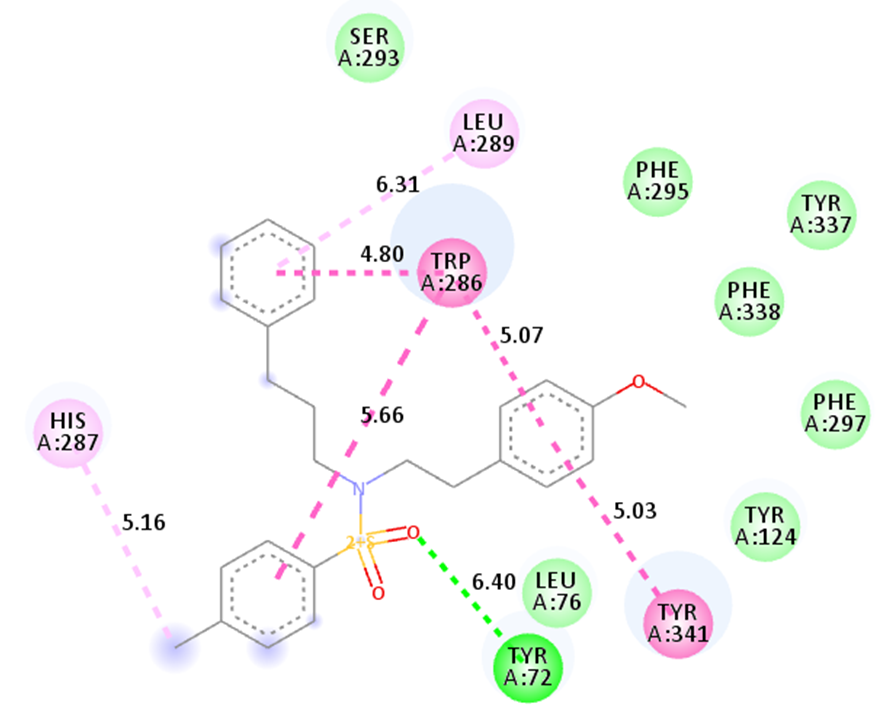


**Figure S10:** Docking complex of 5g


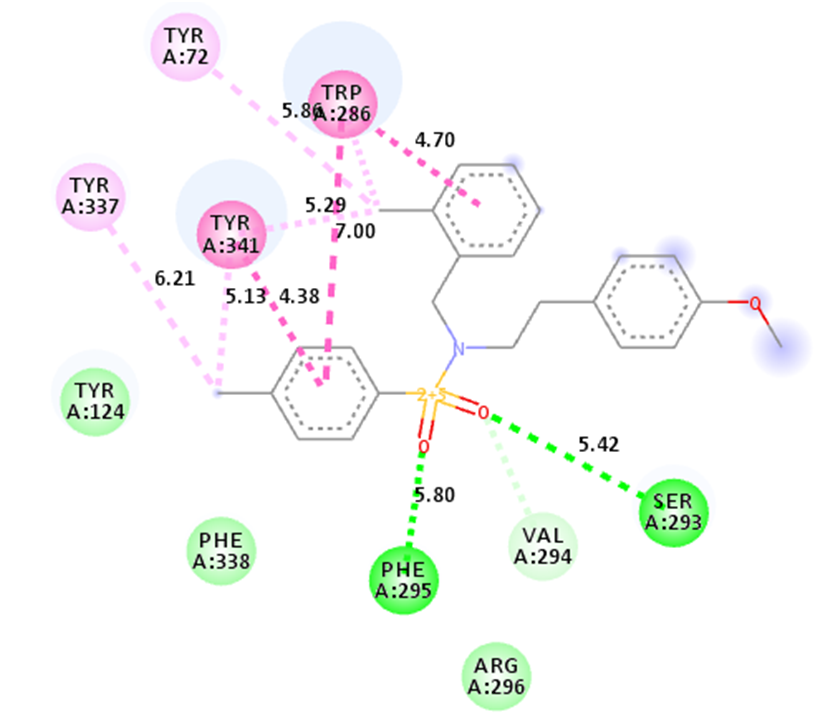


**Figure S11:** Docking complex of 5h


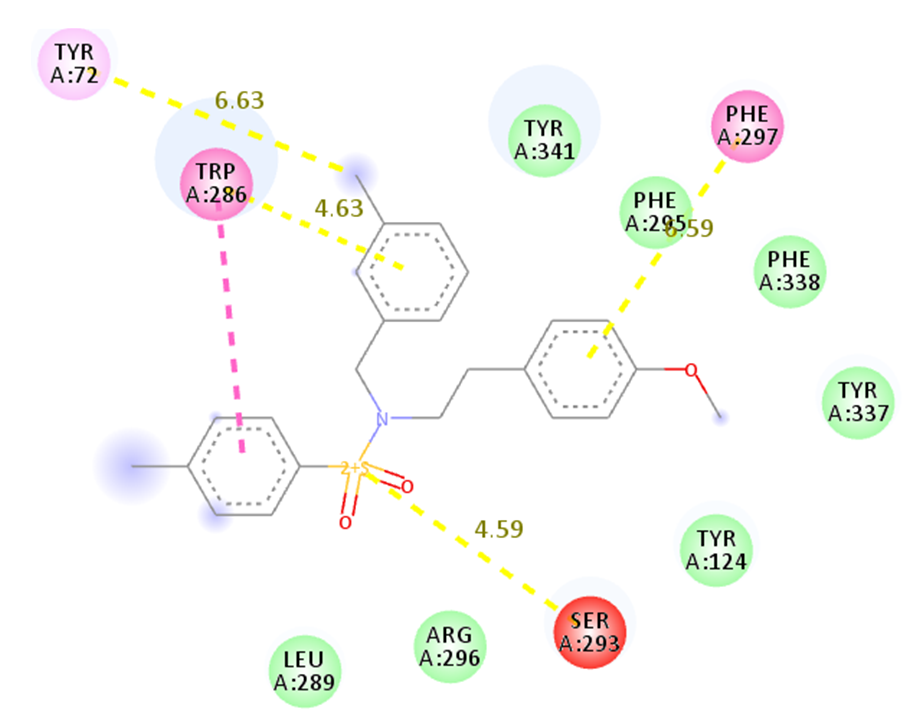


**Figure S12:** Docking complex of 5i


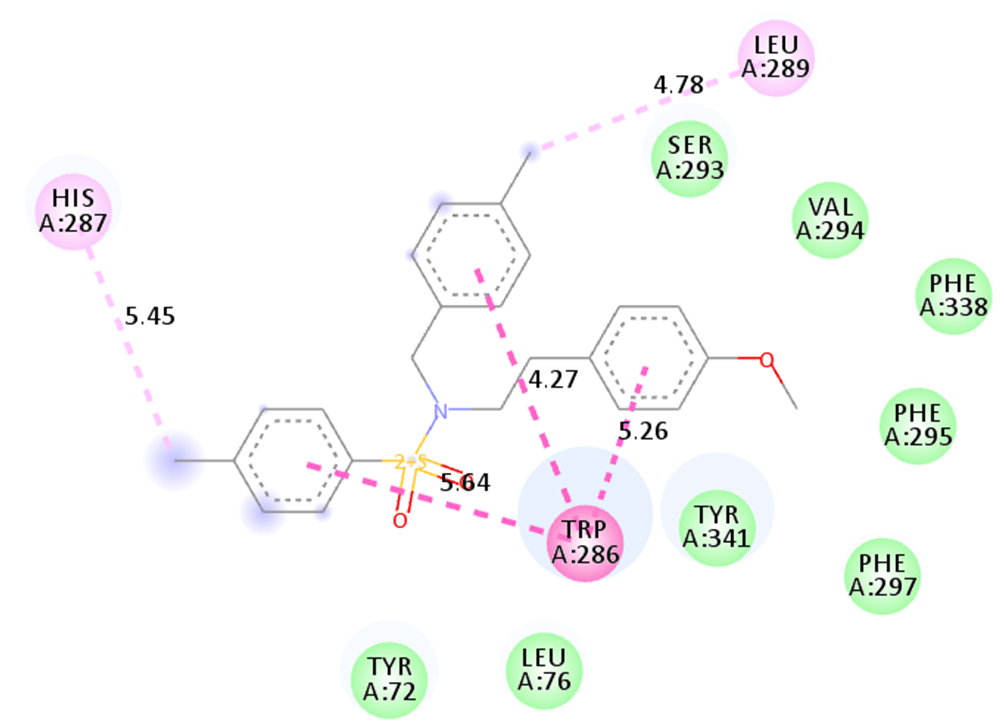


**Figure S13:** Docking complex of 5j
