# Supplementary material for: Synthesis, enzyme inhibitory kinetics mechanism and computational study of N-(4-methoxyphenethyl)-N-(substituted)-4-methylbenzenesulfonamides as novel therapeutic agents for Alzheimer’s disease
Source: PeerJ. 2018 Jun 26;6:e4962. doi: 10.7717/peerj.4962 (PMC6025150; doi:10.7717/peerj.4962)
Supplement: Supplemental Information 2 [file peerj-06-4962-s004.pdf]

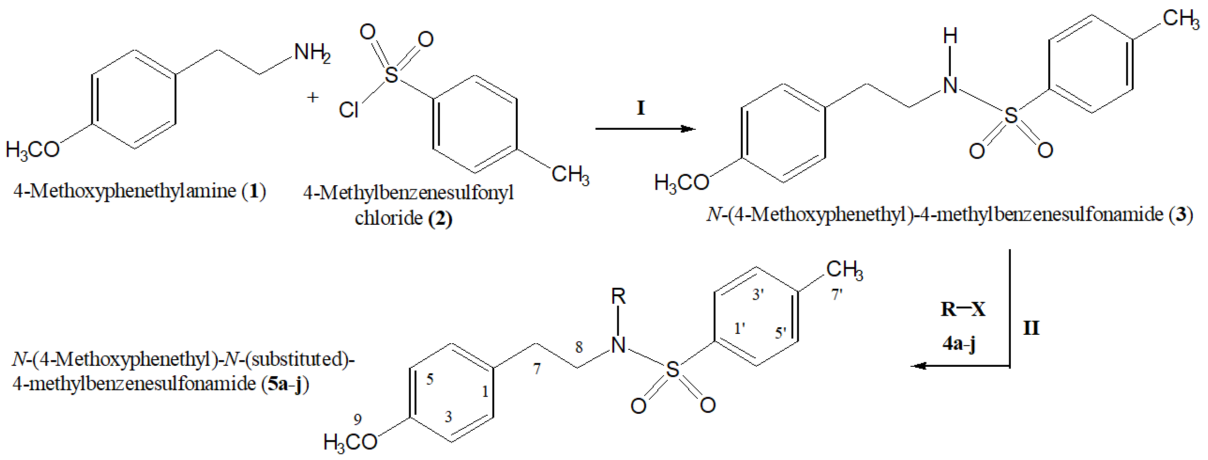

Scheme 1 Outline for the synthesis of different *N*-substituted derivatives, **5a-j**, of *N*-(4-methoxyphenethyl)-4-methylbenzensulfonamide (**3**). Reagents & Conditions: (I) Aq. Na_2CO_3 soln./pH 9-10/stirring at RT for 2-3 hrs. (II) DMF/LiH/stirring at RT for 0.5 hrs for activation/then addition of R-X (**4a-j**) and stirring finally for 4-5 hrs.
